# Activation of TLR Signaling in Sensitization-Recruited Inflammatory Monocytes Attenuates OVA-Induced Allergic Asthma

**DOI:** 10.3389/fimmu.2018.02591

**Published:** 2018-11-19

**Authors:** Chao Huang, Jian Wang, Xiaodong Zheng, Yongyan Chen, Haiming Wei, Rui Sun, Zhigang Tian

**Affiliations:** ^1^Institute of Immunology and The CAS Key Laboratory of Innate Immunity and Chronic Disease, School of Life Sciences and Medical Center, University of Science and Technology of China, Hefei, China; ^2^Hefei National Laboratory for Physical Sciences at Microscale, University of Science and Technology of China, Hefei, China

**Keywords:** allergic asthma, TLR signaling, inflammatory monocytes, Th1-associated cytokines, sensitization

## Abstract

The activation of Toll-like receptor (TLR) signaling is widely reported to be involved in preventing the development of allergic asthma. However, the mechanism of the protective function of TLR signaling remains limited. Here, we studied the mouse model of ovalbumin (OVA)-induced allergic asthma and found that deficiency of TLR signaling or activating TLR signaling with agonist would aggravate or attenuate OVA-induced allergic asthma, respectively, and TLR signaling-mediated protective effect mainly affected the sensitization phase. After OVA/alum sensitization, neutrophils and inflammatory monocytes were recruited into peritoneal cavity and up-regulated TLRs expression. However, adoptive transfer of inflammatory monocytes but not peritoneal macrophages or neutrophils induced allergic symptoms in recipient mice after OVA challenge even without OVA/alum sensitization, and treating the inflammatory monocytes with TLR agonist *in vitro* before transfer could abolish this effect, indicating that recruited inflammatory monocytes played a determinant role in OVA-induced allergic asthma, and activation of TLR signaling in them could attenuate allergic symptoms. Finally, we found that activation of TLR signaling could increase the expression of T-helper (Th) 1-associated cytokines in inflammatory monocytes. Our results suggest that activation of TLR signaling in sensitization-recruited inflammatory monocytes attenuates OVA-induced allergic asthma by promoting the expression of Th1-associated cytokines.

## Introduction

Allergic asthma is an airway inflammatory disease, which is orchestrated by the interaction between genetic factors and environmental antigens, including allergens, microbes, microbial products, et.al. (1). TLRs play a critical role in allergic diseases since they can recognize the environmental antigens to mount a pro- or anti-allergy response (2, 3). The influence of TLRs on the outcome of allergic diseases depends on many factors, including the cell type in which TLR is engaged, the nature of the allergen, the dose of agonist, the route of administration, and the timing of exposure (4–6). For example, intranasal administration of OVA alone in mice cannot induce the airway inflammation, but administration of OVA together with low or high dose of LPS leads to Th2-mediated eosinophilia or Th1/Th17-mediated neutrophilia, respectively (7–11). Thus, TLR signaling could initiate either helpful or harmful responses by modulating the immune system in the context of allergic asthma.

In view of the advantage aspect of TLR signaling in allergic asthma, TLRs are considered as potential therapeutic targets to prevent and treat allergic asthma (12, 13). Studies have shown that activation of TLRs favor to induce Th1/Th17 immune pattern, which can counteract Th2 responses that are predominant in allergic diseases (4). TLRs also can exert immune-regulatory effects through recruiting regulatory T (Treg) cells to the airways (14–17). Thus, manipulation of Th1/Th2 balance or Tregs function by using TLR agonists might be a promising approach to reach the purpose of prevention and treatment. In support of this idea, the agonist of TLR9 has been applied to patients in clinical trials for the treatment of allergic asthma by switching Th2 to Th1 immune responses (18, 19). However, although TLR signaling has displayed the protective function against allergic asthma under some conditions, the cellular and molecular mechanisms of the protective function of TLR signaling in allergic asthma remain controversial or limited. For example, which phase, sensitization or challenge, of OVA-induced allergic asthma could be affected by TLR signaling? And which type of inflammatory cells would be regulated by TLR signaling and finally determined the outcome of the allergic asthma?

In this study, we found that TLR signaling displayed a protective function against OVA-induced allergic asthma, and it exerted this function at the sensitization phage of OVA-induced allergic asthma. Further studies showed that recruited inflammatory monocytes in peritoneal cavity after OVA/alum sensitization played a critical role to determine the severity of OVA-induced allergic asthma. Recruited inflammatory monocytes up-regulated TLR expression, and activation of TLR signaling with agonists in inflammatory monocytes could attenuate OVA-induced allergic asthma by promoting the expression of Th1-associated cytokines.

## Materials and methods

### Animals

Six to eight weeks old mice were used in this study. C57BL/6 (B6, CD45.2^+^) mice were purchased from the Shanghai Laboratory Animal Center (SLAC, Shanghai, China). CD45.1^+^ mice (B6 background) were obtained from the Jackson Laboratory. *Tlr2*^−/−^, *tlr4*^−/−^, and *tlr9*^−/−^ (B6 background) mice were gifts from Dr. Shaobo Su (Sun Yat-sen University, Guangdong, China). All mice were housed in a specific pathogen-free facility.

### Asthma model and TLR agonist treatment

For establishing the asthma model, mice were immunized at days 0 and 7 i.p., with 100 μg of OVA (grade V; Sigma–Aldrich, Saint Louis, MO, USA) in 100 μL of sterile saline and adsorbed in 50 μL of Imject alum (Thermo Scientific, Waltham, MA, USA). At days 14, 15, and 16, mice were administered i.n., with 100 μg of OVA in 50 μL of sterile saline after anesthetized with sodium pentobarbital (50 μg/g body weight, i.p., Merck, Whitehouse Station, NJ, USA). Mice were harvested and analyzed at day 21. For the experiment of TLR activation, WT mice were injected (i.p., or i.n.) with 2.5–10 μg of Pam3CSK4 (InvivoGen, San Diego, CA, USA) or 25 μg of CpG (Sangon Biotech, Shanghai, China) at indicated time points.

### Cell isolation

To obtain cells in pulmonary alveoli, we performed bronchoalveolar lavage as described before (20, 21) with slight modifications. The whole lung was perfused through the trachea with 1 mL of phosphate-buffered saline (PBS) containing 5 mM of EDTA for 4 times. The bronchoalveolar lavage fluid (BALF) was collected and cell pellets were pooled. To obtain single-lung-cell suspensions, lungs were minced and digested in RPMI-1640 medium containing 0.1% collagenase I (Sigma–Aldrich) and 5% fetal bovine serum (Gibco) for 60 min at 37°C. After filtration through a 200-gauge steel mesh, the medium was centrifuged and the red blood cells (RBCs) were removed by RBC lysis buffer (BioLegend). The cells were washed and collected for further experiments. For the isolation of peritoneal cells, mice were injected i.p., with 5 mL of sterile saline containing 5 mM of EDTA. The abdomen was rubbed gently for 2 min, and the lavage fluids were collected. Lavage fluids were centrifuged, and cell pellets were collected. Cell numbers were counted by Countstar® (Shanghai Ruiyu Biotech, China).

### Enzyme-linked immunosorbent assay (ELISA)

For analyses of levels of cytokines and antibody in BALF, the lung was perfused through the trachea with 1 mL of PBS containing 5 mM of EDTA. Lavage fluids were centrifuged, and the supernatants were collected. Levels of IL-4, IL-5, and IgE in BALF and serum were measured by using ELISA kits from Dakewe Biotech (Shenzhen, China) according to the manufacturer's instructions.

### Flow cytometry

Flow cytometry analysis were performed as reported before (22) with slight modifications. For surface staining, single cells were incubated with fluorescein-labeled monoclonal antibodies for 30 min at 4°C after blockade of the Fc receptor, then washed twice and analyzed. For IL-13 staining, cells were stimulated with 50 ng/mL of PMA, 1 μg/mL of ionomycin and 10 μg/mL of monensin (all from Sigma–Aldrich) for 4 h at 37°C. After staining of cell-surface markers, cells were fixed and permeabilized with a Foxp3 Staining Buffer Set (BioLegend) according to the manufacturer's protocols and then stained with anti-mouse IL-13 or isotype control overnight at 4°C. Samples were analyzed by BD LSRII or LSRFortessa system (BD Biosciences, Franklin Lakes, NJ, USA) and FlowJo software (Tree Star, Ashland, OR, USA). Antibodies and isotype controls were purchased form BD Biosciences, BioLegend (San Diego, CA, USA), or eBioscience (San Diego, CA, USA) (Supplementary Table 1).

### Cell sorting and transfer

The mice were immunized with 100 μg of OVA in 100 μL of sterile saline and adsorbed in 50 μL of Imject alum. The peritoneal lavage fluids (PLF) were collected, and pMφ, inflammatory monocytes and neutrophils in PLF were purified by FACSAria II cell sorter (BD Biosciences). Purified cells were resuspended in saline and injected i.p., into the recipient mice at indicated time points.

### RNA isolation and quantitative PCR

Total RNA was extracted from 2–5 × 10^5^ purified inflammatory monocytes using TRIzol® Reagent (Invitrogen, Carlsbad, CA, USA). cDNA was synthesized with SuperScript III Reverse Transcriptase (Invitrogen) according to the manufacturer's protocols. Then, gene expression was analyzed using SYBR™ Premix Ex Taq kit (TaKaRa Bio, Kusatsu, Japan) and quantified using the ^ΔΔ^Ct method. The primers used in this study were shown as following: GAPDH, 5′*-*tgcccagacatcatccctg-3′ (Forward) and 5′-tcagatccacgacggacaca-3′(Reverse); IL-6, 5′-taacagataagctggagtc-3′ (Forward) and 5′-taggtttgccgagtaga-3′ (Reverse); TNF-α, 5′-tctcattcctgcttgtggc-3′ (Forward) and 5′-cacttggtggtttgctacg-3′ (Reverse); IL-12, 5′- gcagcgtgggagtgggatgtg-3′(Forward) and 5′-gggcatcgggagtccagtcca-3′ (Reverse); IFN-γ, 5′-gccatcagcaacaacataagc-3′ (Forward) and 5′-gagctcattgaatgcttggc-3′ (Reverse); IL-4, 5′-caacccccagctagttgtca-3′(Forward) and 5′- cgttgctgtgaggacgtttg-3′(Reverse); IL-5, 5′-agcaatgagacgatgaggct-3′(Forward) and 5′- gtacccccacggacagtttg-3′(Reverse); IL-13, 5′- tcttgcttgccttggtggtc-3′(Forward) and 5′- ggggagtctggtcttgtgtg-3′(Reverse). All primers were synthesized by Sangon Biotech.

### Histology

Lung tissues without lavage were fixed in 10% (*v/v*) formalin for >24 h and embedded in paraffin. Sections (6 μm) were stained with hematoxylin and eosin. Peribronchial and perivascular inflammation was assessed by a semi-quantitative scoring system as described before (23). The lung inflammation was scored following the scale: 0, normal; 1, few cells; 2, a ring of inflammatory cells one cell layer deep; 3, a ring of inflammatory cells two to four cells deep; and 4, a ring of inflammatory cells of more than four cells deep.

### Statistical analysis

All data were expressed as mean ± SEM and analyzed using the Student's two-tailed *t*-test. A value of *P* < 0.05 was considered statistically significant.

## Results

### TLR signaling plays a protective role in OVA-induced allergic asthma

**Figure 1:**
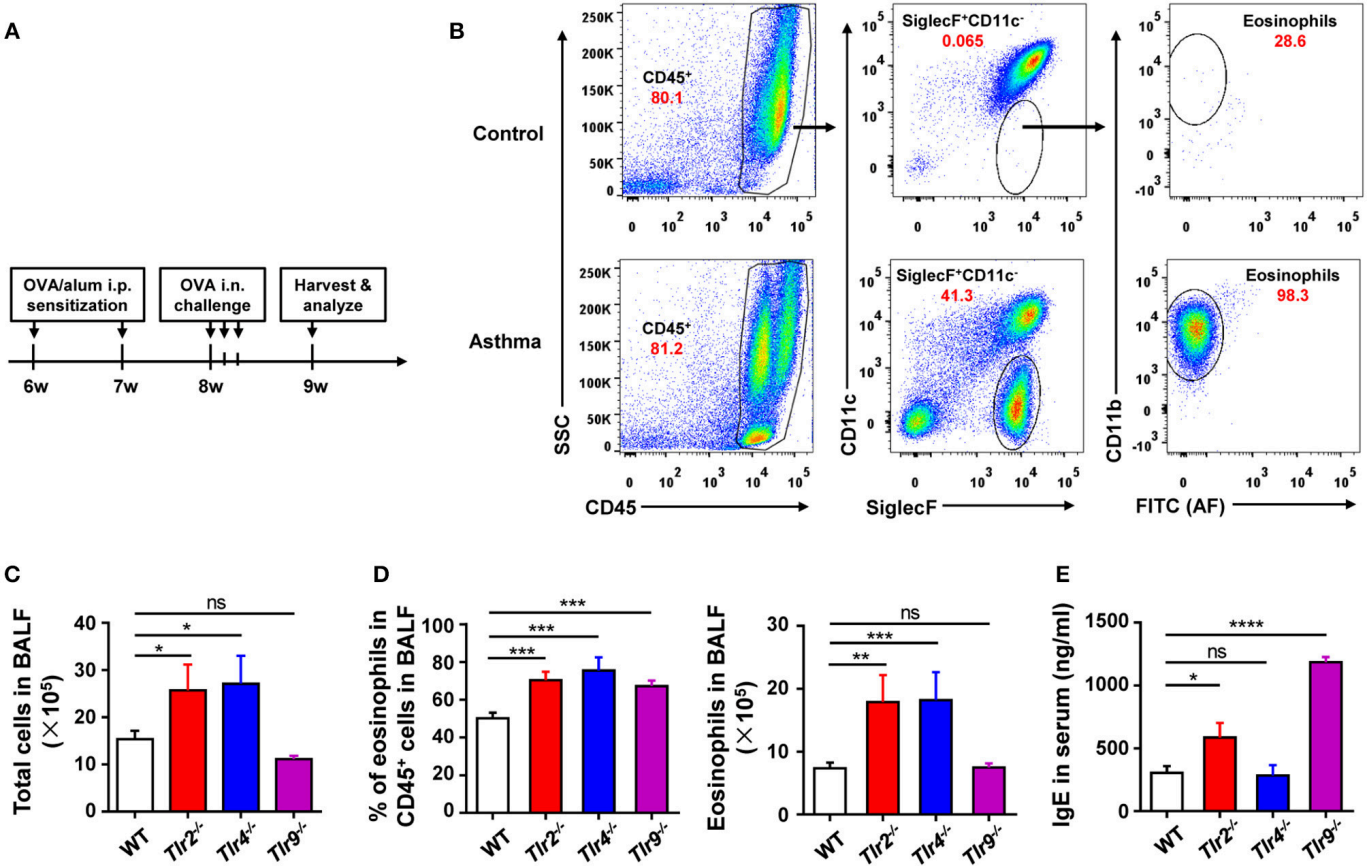
Deficiency of TLR signaling aggravates OVA-induced allergic asthma in mice. **(A)** Procedure of OVA-induced allergic asthma in mice: Mice were treated i.p., with OVA/alum for sensitization at 6- and 7-weeks-old and challenged i.n., with OVA at 9-weeks-old for 3 consecutive days. All mice were harvested and analyzed 1 week after OVA challenge. **(B)** Eosinophils in BALF from asthmatic and control mice were analyzed by FACS. Eosinophils were identified as CD45^+^SiglecF^+^CD11b^+^CD11c^−^ cells. **(C–E)** 6-weeks-old *tlr2*^−/−^, *tlr4*^−/−^, *tlr9*^−/−^, and control WT mice were sensitized i.p., with OVA/alum and challenged i.n., with OVA to induce allergic asthma. Total cells in BALF were counted **(C)**, the frequency and number of eosinophils in BALF were analyzed by FACS **(D)**, and the level of IgE in serum was analyzed by ELISA **(E)**. Data are representative of three or more independent experiments with ≥4 mice per group. Data are the mean ± SEM. *P < 0.05, **P < 0.01, ***P < 0.0001, ****P < 0.00001, ns, not significant.

**Figure 2:**
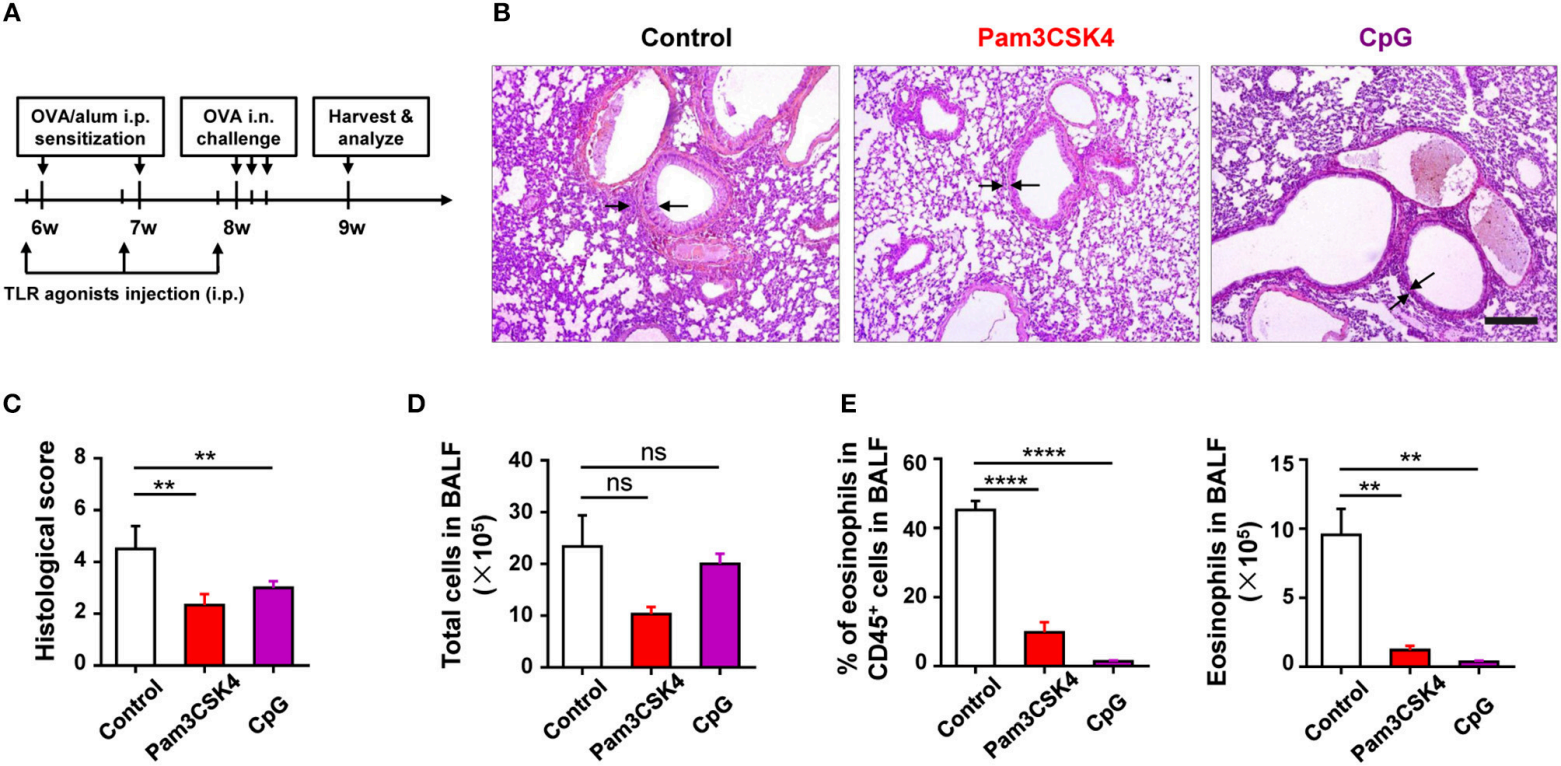
Activation of TLR signaling protects mice against OVA-induced allergic asthma. **(A–E)** 6-weeks-old WT mice were treated i.p., with 10 μg of Pam3CSK4 or 25 μg of CpG 1 day before OVA/alum sensitization and OVA challenge in the mouse model of OVA-induced allergic asthma. All mice were harvested and analyzed 1 week after OVA challenge **(A)**. Histologic sections of lungs from each group were analyzed by H&E. Pictures show representative samples of 4–6 mice/group. Scale bar = 200 μm **(B)**. Histological scores for assessment of lung injury were shown **(C)**. Total cells in BALF were counted **(D)**, and the frequency and number of eosinophils in BALF were analyzed by FACS **(E)**. Data are representative of three or more independent experiments with ≥4 mice per group. Data are the mean ± SEM. **P < 0.01, ****P < 0.00001, ns, not significant.

To find out the cellular and molecular mechanisms of the protective function of TLR signaling in allergic asthma, we established the mouse model of OVA-induced allergic asthma by peritoneal sensitization using OVA/alum and intranasal challenge with OVA (Figures 1A,B), which is the most widely used mouse model of allergic asthma (24). To confirm if TLR signaling had a protective role in our mouse model, we established OVA-induced allergic asthma in *tlr2*^−/−^, *tlr4*^−/−^, and *tlr9*^−/−^ mice, and WT mice were used as the control. The results showed that, compared with WT mice, *tlr2*^−/−^, and *tlr4*^−/−^ mice exhibited increased infiltration of leukocytes, especially asthma-related eosinophils, in alveoli (Figures 1C,D), and IgE level in serum was increased significantly in *tlr2*^−/−^ and *tlr9*^−/−^ mice after allergic asthma establishment (Figure 1E). Activation of various TLRs by agonists has been reported to prevent OVA-induced allergic asthma (25–27). To confirm this protective effect of TLR agonists in our mouse model, we treated WT mice intraperitoneally (i.p.) with the TLR2 agonist Pam3CSK4 or TLR9 agonist CpG (Figure 2A). The treatment of TLR agonists could significantly reduce lung injury (Figures 2B,C) and the number of infiltrating leukocytes and eosinophils in alveoli after allergic asthma establishment (Figures 2D,E). Therefore, these results indicated that activation of TLR signaling protected against OVA-induced allergic asthma in our mouse model, which was consistent with previous reports.

### TLR signaling attenuates OVA-induced allergic asthma by affecting sensitization

**Figure 3:**
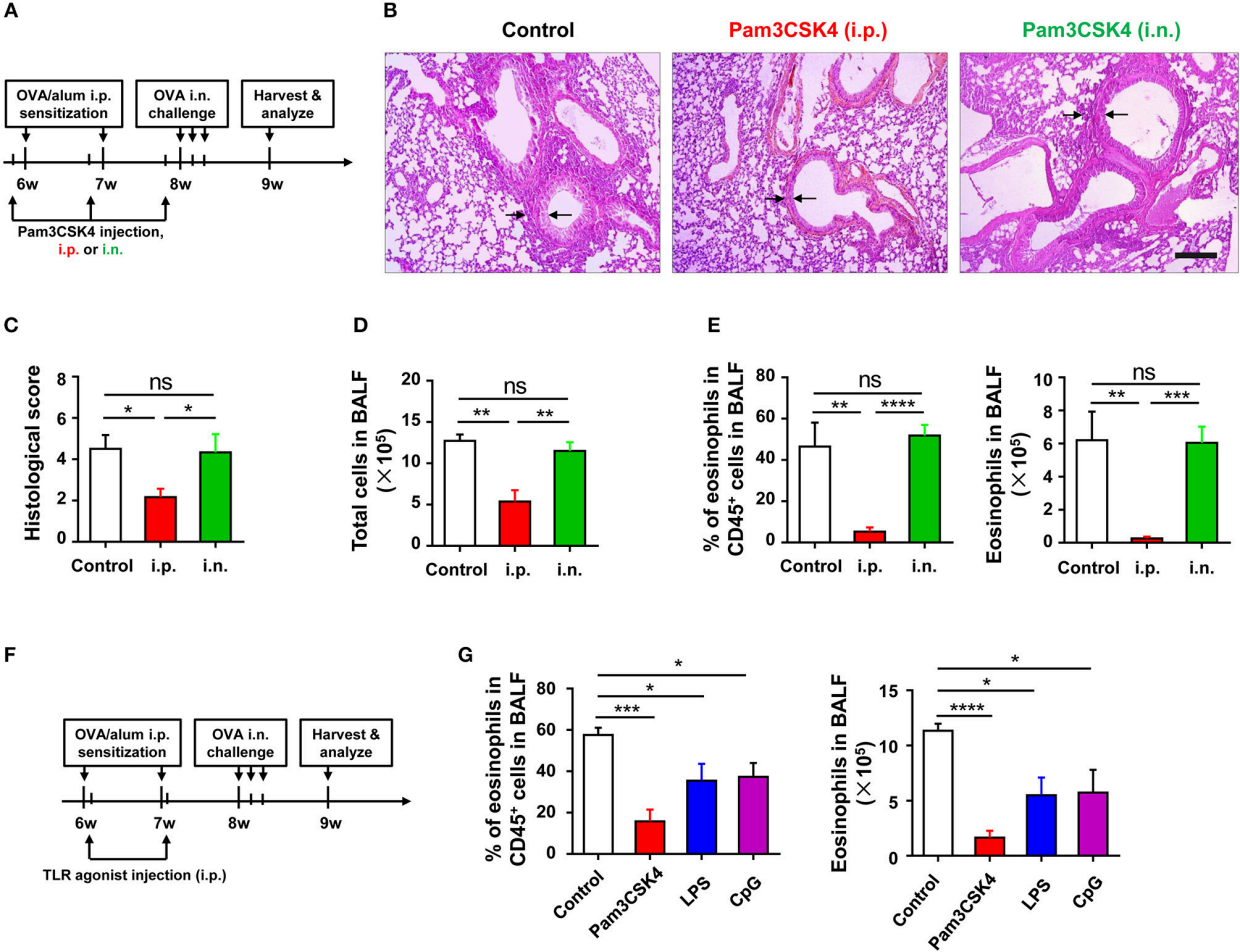
TLR exerts protective function at sensitization phase of OVA-induced allergic asthma. **(A–E)** 6-weeks-old WT mice were treated i.p., or i.n., with 10 μg of Pam3CSK4 1 day before OVA/alum sensitization and OVA challenge in the mouse model of OVA-induced allergic asthma. All mice were harvested and analyzed 1 week after OVA challenge **(A)**. Histologic sections of lungs from each group were analyzed by H&E. Pictures show representative samples of 4–6 mice/group. Scale bar = 200 μm **(B)**. Histological scores for assessment of lung injury were shown **(C)**. Total cells in BALF were counted **(D)**, and the frequency and number of eosinophils in BALF were analyzed by FACS **(E)**. **(F–G)** 6-weeks-old WT mice were treated i.p., with 2 μg of Pam3CSK4, LPS, or CpG 4 h after OVA/alum sensitization in the mouse model of OVA-induced allergic asthma, respectively. Mice injected i.p., with saline were used as control. All mice were harvested and analyzed 1 week after OVA challenge **(F)**. The frequency and number of eosinophils in BALF were analyzed by FACS **(G)**. Data are representative of three or more independent experiments with ≥4 mice per group. Data are the mean ± SEM. *P < 0.05, **P < 0.01, ***P < 0.0001, ****P < 0.00001, ns, not significant.

To identify which phase (e.g., sensitization or challenge) of OVA-induced allergic asthma could be affected by TLR signaling, we treated WT mice with Pam3CSK4 by peritoneal and intranasal routes, respectively (Figure 3A). We found that only treating mice i.p., with Pam3CSK4 protected against OVA-induced allergic asthma, characterized by reduced lung injury and less inflammatory cells, especially eosinophils, infiltration in alveoli when compared with WT control mice, but treating mice intranasally (i.n.) with Pam3CSK4 had no this protective effect (Figures 3B–E), implying that TLR agonist treatment might mainly influence the sensitization response in the peritoneal cavity. To prove this hypothesis, different from the mode of administration in Figure 3A, we only treated mice i.p., with Pam3CSK4 at the sensitization stage, and the Pam3CSK4 was i.p., injected after OVA/alum sensitization (Figures 3F). The results showed that Pam3CSK4 injection only at sensitization stages showed equivalent protection compare to the mode of administration in Figures 3A,G. Thus, these results indicated that TLR signaling-mediated protective effect mainly affect the sensitization phase of OVA-induced allergic asthma.

### Innate inflammatory cells are recruited after sensitization and up-regulate TLR expression

**Figure 4:**
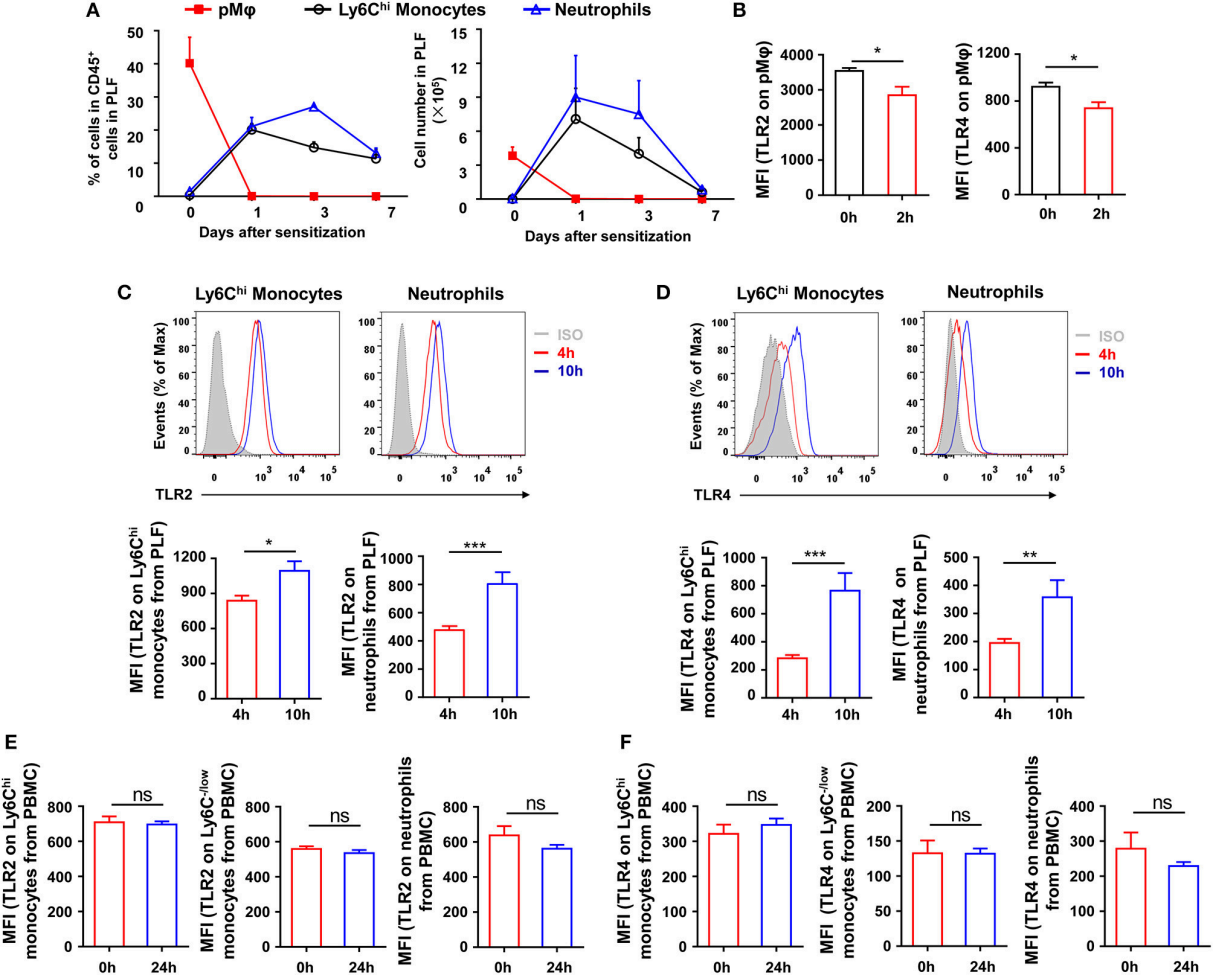
Inflammatory cells are recruited and upregulate TLRs expression after sensitization. **(A–B)** 6-weeks-old WT mice were treated i.p., with OVA/alum. The frequency and number of pMφ (CD45^+^F4/80^hi^CD11b^hi^), inflammatory monocytes (CD45^+^F4/80^lo^Ly6C^hi^CD11b^+^), and neutrophils (CD45^+^Ly6G^hi^CD11b^hi^) in PLF were analyzed by FACS at the indicated time points after OVA/alum treatment **(A)**. The expressions of TLR2 and TLR4 on pMϕ were analyzed by FACS 0 and 2 h after OVA/alum treatment **(B)**. **(C–D)** The expressions of TLR2 **(C)** and TLR4 **(D)** on inflammatory monocytes and neutrophils in PLF were analyzed by FACS 4 and 10 h after OVA/alum treatment. **(E–F)** The expressions of TLR2 **(E)** and TLR4 **(F)** on Ly6C^hi^ monocytes, Ly6C^−/low^ monocytes, and neutrophils in blood were analyzed by FACS 0 and 24 h after OVA/alum treatment. Data are representative of three or more independent experiments with ≥4 mice per group. Data are the mean ± SEM. *P < 0.05, **P < 0.01, ***P < 0.001, ns, not significant.

To explore which type of inflammatory cells in the peritoneal cavity could be regulated by TLR signaling at the stage of sensitization, we first monitored the dynamic change of inflammatory cells in the peritoneal cavity after OVA/alum sensitization (Supplementary Figure 1 in Supplementary Material). Inflammatory monocytes and neutrophils were recruited and increased in the peritoneal cavity, along with the decrease of pMφ, after OVA/alum sensitization (Figure 4A). Analyses of TLRs expression on cells showed that expression of TLR2 and TLR4 decreased on pMφ (Figure 4B) but increased on recruited inflammatory monocytes and neutrophils over time after OVA/alum sensitization (Figures 4C,D). Meanwhile, there were no changes of TLR2 and TLR4 expressions on monocytes and neutrophils in blood after OVA/alum sensitization (Figures 4E,F), which means that the microenvironment in peritoneal cavity after OVA/alum sensitization promoted the expression of TLR2 and TLR4. Our previous study found that pMφ almost could not be found in the peritoneal cavity 4 hours after OVA/alum sensitization (28). However, we also found that treating mice i.p., with Pam3CSK4, LPS, or CpG 4 h after sensitization also could significantly attenuate OVA-induced allergic asthma (Figures 3A,B). Thus, activation of TLR signaling in inflammatory monocytes and/or neutrophils (but not pMφ) might be able to attenuate OVA-induced allergic asthma.

**Figure 5:**
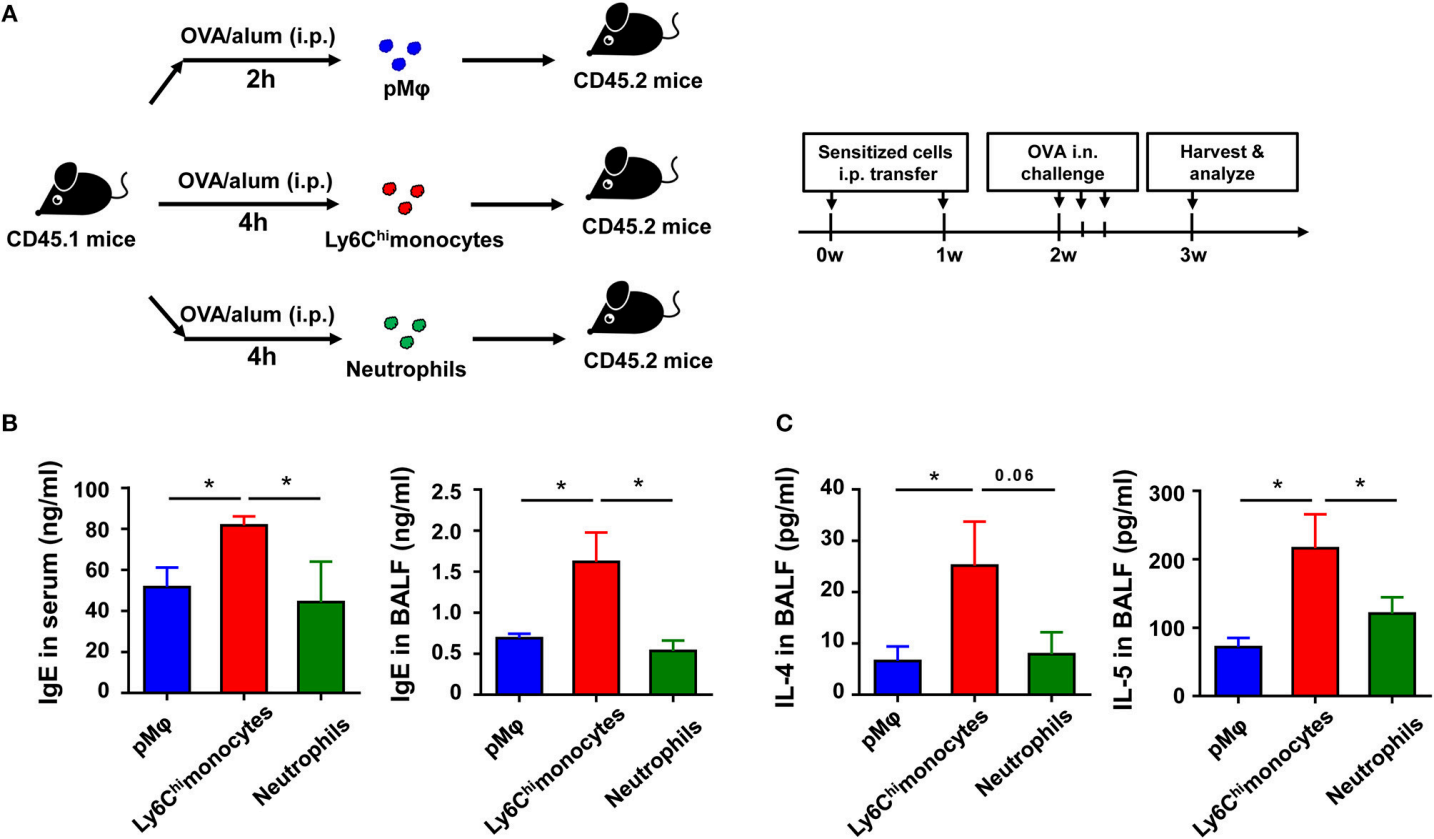
Recruited inflammatory monocytes are crucial for allergic response in OVA-induced allergic asthma. **(A–C)** CD45.1 mice were treated i.p., with OVA/alum, and PLF were harvested 2 h after treatment to purify pMφ or 4 h after treatment to purify inflammatory monocytes and neutrophils by sorting. 1.5 × 10^5^ of each cell subset were transferred i.p., into CD45.2 recipient mice immediately after sorting once a week for 2 weeks, respectively. The CD45.2 recipient mice were challenged i.n., with OVA 1 week after the second transfer. All CD45.2 recipient mice were harvested and analyzed 1 week after OVA challenge **(A)**. The levels of IgE in serum and BALF were analyzed by ELISA **(B)**, and the levels of IL-4 and IL-5 in BALF from each group were analyzed by ELISA **(C)**. Data are representative of three or more independent experiments with ≥4 mice per group. Data are the mean ± SEM. *P < 0.05, ns, not significant.

To further determine which type of cells, inflammatory monocytes and/or neutrophils, play a critical role in OVA-induced allergic asthma, we purified pMφ, inflammatory monocytes, and neutrophils from OVA/alum-sensitized CD45.1 WT mice (Supplementary Figure 2 in Supplementary Material) and then adoptively transferred them to CD45.2 recipient mice, respectively (Figure 5A). All three cells transfer could induce allergic symptoms in recipient mice after OVA challenge even without OVA/alum sensitization, but transfer of inflammatory monocytes could induce more severe asthma in recipient mice than transfer of pMφ or neutrophils (Figures 5B,C). Taken together, these results suggested that activation of TLR signaling in inflammatory monocytes might be the main reason for the protective function of TLR signaling in OVA-induced allergic asthma.

### Activation of TLR signaling in inflammatory monocytes attenuates allergic th2 response after OVA challenge

**Figure 6:**
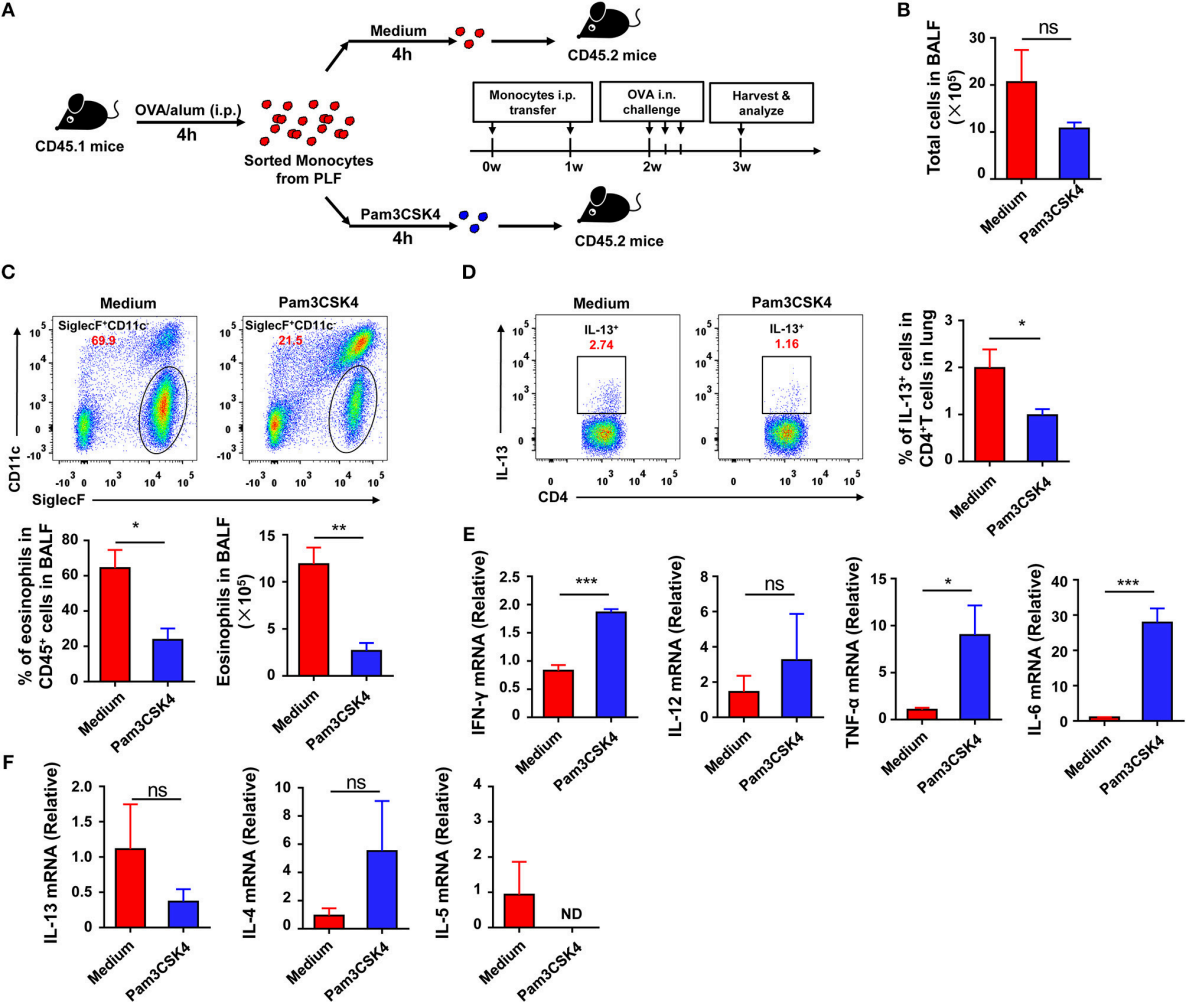
Activation of TLR signaling in inflammatory monocytes attenuates OVA-induced allergic asthma by increasing Th1 cytokines. **(A–F)** CD45.1 mice were treated i.p., with OVA/alum, and PLF were harvested 4 h after treatment to purify inflammatory monocytes by sorting. 1.5 × 10^5^ inflammatory monocytes were transferred i.p., into CD45.2 recipient mice after co-culturing with medium or 10 μg/mL of Pam3CSK4 for 4 h once a week for 2 weeks. CD45.2 recipient mice were challenged i.n., with OVA 1 week after the second transfer. All CD45.2 recipient mice were harvested and analyzed 1 week after challenge **(A)**. Total cells in BALF were counted **(B)**. The frequency and number of eosinophils (Gating on CD45^+^ single cells) in BALF from each group were analyzed by FACS **(C)**. The frequency of Th2 cells (Gating on CD3^+^CD4^+^ cells) in total CD4^+^ T cells from lung was analyzed by FACS **(D)**. Expressions of Th1-type cytokines (IFN-γ, TNF-α, IL-6, and IL-12) **(E)** and Th2-type cytokines (IL4, IL-5, IL-13) **(F)** in purified inflammatory monocytes 4 h after medium or Pam3CSK4 treatment *in vitro* were analyzed by real-time PCR. Data are representative of more than two independent experiments with ≥4 mice per group. Data are the mean ± SEM. *P < 0.05, **P < 0.01, ***P < 0.001, ns, not significant, ND, not detected.

To explore if the recruited inflammatory monocytes could be regulated by TLR signaling and if this effect could affect subsequent asthma disease, we treated the purified inflammatory monocytes with the TLR2 agonist Pam3CSK4 *in vitro* before transfer into recipient mice (Figure 6A). The result showed that Pam3CSK4-treated inflammatory monocytes induced milder allergic symptoms in recipient mice after OVA challenge when compared with Pam3CSK4-untreated inflammatory monocytes (Figures 6B–D). It has been reported that allergic asthma is an allergen-induced Th2 inflammatory disease, and activation of TLR signaling prevents OVA-induced allergic asthma in mice through promoting Th1 response (26). Consistent with previous reports, recruited inflammatory monocytes significantly up-regulated the expression of Th1-associated cytokines, including interferon (IFN)-γ, tumor necrosis factor (TNF)-α, and interleukin (IL)-6, after stimulating with TLR2 agonist Pam3CSK4 *in vitro* (Figure 6E). Furthermore, there was no significant change of the expression of IL-13 and IL-4 after stimulating with TLR2 agonist Pam3CSK4 *in vitro*, but IL-5 expression was down-regulated after stimulation (Figure 6F). Taken together, these results suggested that activation of TLR signaling in recruited inflammatory monocytes could promote the expression of Th1-associated cytokines to inhibit allergen-induced Th2 response and finally protected mice against OVA-induced allergic asthma.

## Discussion

Allergic asthma has already became a significant global public health problem with an increasing rate of prevalence and occurrence over the past decades, so it is urgent to find efficient ways to prevent and treat this disease (29). Increasing evidences support that activation of TLR signaling with agonists would be a potential way to control the allergic asthma (18). In this study, we found that, consistent with previous reports, activation of TLR signaling with agonists could protect mice against OVA-induced allergic asthma. Exploring the mechanism found that TLR signaling played the protective function at the sensitization but not challenge phase of OVA-induced allergic asthma, and activation of TLR signaling in sensitization recruited-inflammatory monocytes significantly attenuated allergic symptoms since they up-regulated the expression of Th1-association cytokines, including IFN-γ, IL-6, and TNF-α, after stimulation with TLR agonist.

Up to now, 12 TLRs have been identified. Among them, TLR3, 7, 8, and 9 are localized within endosomal compartments of cells, and the others are found on the plasma membrane (30). The role of TLRs in the development of allergic asthma and the possibility of applying TLRs as a target for asthma therapy have been widely studied (2, 3, 31). However, the goal of applying TLRs as a target for asthma therapy is not easy to achieve, because the signaling pathways of TLRs have a very complex network (32, 33). In our study, although *tlr2*^−/−^*, tlr4*^−/−^, and *tlr9*^−/−^ mice all displayed more severe disease compared with WT mice after allergic asthma establishment, the parameters of allergic symptom, including total cell number, the number of eosinophil and IgE level, in these mice displayed difference. Compared with WT mice, *tlr2*^−/−^, *tlr4*^−/−^ but not *tlr9*^−/−^ mice exhibited increased infiltration of eosinophils in alveoli after allergic asthma establishment. IgE levels in serum were increased significantly in *tlr2*^−/−^*, tlr9*^−/−^ but not in *tlr4*^−/−^ mice after allergic asthma establishment. Thus, different TLRs might influence the development of allergic asthma with different signaling pathways.

Although the manipulation of TLRs to control and treat allergic asthma has received extensive attention, some studies showed that, in contrast with the protective function of TLRs, TLRs played a harmful role in asthma development (2, 34). Thus, TLRs play the role like a double-edged sword in the development of allergic asthma. The results from human and mouse studies indicated that the influence of TLRs on allergic asthma is affected by many factors, such as the cell type in which TLR is engaged and the route of administration (4). Our previous study found that TLRs on peritoneal macrophage promotes the allergic asthma by maintaining the NLRP3/IL-1β signaling (28), but in this study, activation of TLRs signaling in sensitization-recruited monocytes could attenuate allergic asthma by producing Th1-associated cytokines. Furthermore, in this study, we found that only treating mice i.p., with Pam3CSK4 protected against OVA-induced allergic asthma, but treating mice i.n., with Pam3CSK4 had no this protective effect, which means that, at least in the mouse model that we used in this study, TLR signaling-mediated protective effect mainly affect the sensitization phase of OVA-induced allergic asthma.

Our previous studies found that NLRP3/IL-1β signaling in peritoneal macrophages promoted OVA-induced allergic asthma through recruiting inflammatory monocytes and neutrophils into peritoneal cavity after OVA/alum sensitization, and inflammatory monocytes played a dominant role to determine the outcome of OVA-induced allergic asthma (28), which was further confirmed in this study. These results indicated that inflammatory monocytes played a role to promoted Th2 response in OVA-induced allergic asthma. However, in current study, activation of TLR signaling by agonists in inflammatory monocytes could attenuate OVA-induced allergic asthma by inducing the expression of Th1-associated cytokines. We wondered what factors could influence the expression of Th1- or Th2-associated cytokines in inflammatory monocytes? The dose of TLRs agonist might be a reason for it. As reported before, intranasal administration of OVA together with a low dose of LPS leads to a Th2-mediated inflammatory response, but administrating together with a high dose of LPS results in a Th1/Th17-mediated inflammatory response (7–9). Thus, a weak stimulation to TLRs would apt to induce a Th2 response, but a strong stimulation would prefer a Th1/Th17 response.

According to WHO estimates, 235 million people are suffering from asthma, and the number is increasing over year (35). Although increasing evidences support that TLRs would be potential target to prevent and treat allergic asthma, there are still some critical questions that need to be answered. For examples, how to choose an optimal dose of TLRs agonist? Which is the best route to administrate the TLRs agonist? How to limit the action of TLRs agonist in local but not in system? Our findings provide some clues to answer these questions, but more precise mechanisms and deeper information are needed.

## Ethics statement

All of the animal protocols were approved by Local Ethics Committee for Animal Care and Use at University of Science and Technology of China (Authorization number: USTCACUC1601003, Hefei, China).

## Author contributions

CH designed and performed the experiments, analyzed and interpreted the data. RS and HW established techniques of FACS and histology and interpreted the data. XZ and YC assisted with data interpretation. ZT provided strategic planning, conceived the project, and interpreted the data. JW supervised the project, provided crucial ideas, and assisted with data interpretation. CH wrote the manuscript with JW and ZT.

### Conflict of interest statement

The authors declare that the research was conducted in the absence of any commercial or financial relationships that could be construed as a potential conflict of interest.

## Abbreviations

BALF: bronchoalveolar lavage fluid
Ig: immunoglobulin
OVA: ovalbumin
PLF: peritoneal lavage fluid
pMφ: peritoneal macrophages
Th: T-helper
TLRs: Toll-like receptors
TNF-α: tumor necrosis factor α
Interferon: IFN.
