# Supplementary material for: Activation of TLR Signaling in Sensitization-Recruited Inflammatory Monocytes Attenuates OVA-Induced Allergic Asthma
Source: Front Immunol. 2018 Nov 19;9:2591. doi: 10.3389/fimmu.2018.02591 (PMC6252340; doi:10.3389/fimmu.2018.02591)
Supplement: Supplementary file 1 [file Data_Sheet_1.PDF]

Supplementary Material

Activation of TLR Signaling in Sensitization-recruited Inflammatory Monocytes Attenuates OVA-induced Allergic Asthma

Chao Huang[1], Jian Wang[1*], Xiaodong Zheng[1], Yongyan Chen[1], Haiming Wei[1,2], Rui Sun[1,2], and Zhigang Tian[1,2*]

[1]Institute of Immunology and The CAS Key Laboratory of Innate Immunity and Chronic Disease, School of Life Sciences and Medical Center, University of Science and Technology of China, Hefei, Anhui 230027, China.

[2]Hefei National Laboratory for Physical Sciences at Microscale, University of Science and Technology of China, Hefei, Anhui 230027, China.

[*]Correspondence: Z.T. (tzg@ustc.edu.cn) or J.W. (ustcwj@mail.ustc.edu.cn).

1 Supplementary Figures

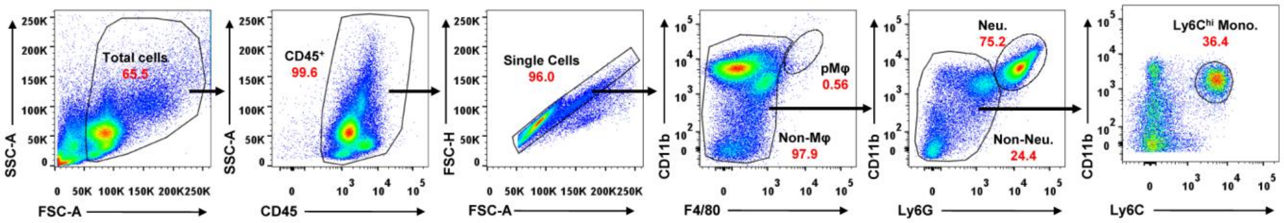

Supplementary Figure 1 | Gating strategy for PLF in flow cytometry. WT B6 mice were i.p. injected with OVA/alum, and cells in PLF were collected 4 hours later for FACS analysis. Peritoneal macrophages in PLF were gated as $CD45^{+}F4/80^{hi}CD11b^{hi}$ cells, inflammatory monocytes in PLF were gated as $CD45^{+}F4/80^{lo}Ly6C^{hi}CD11b^{+}$ cells, and neutrophils in PLF were gated as $CD45^{+}Ly6G^{hi}$ $CD11b^{hi}$ cells.

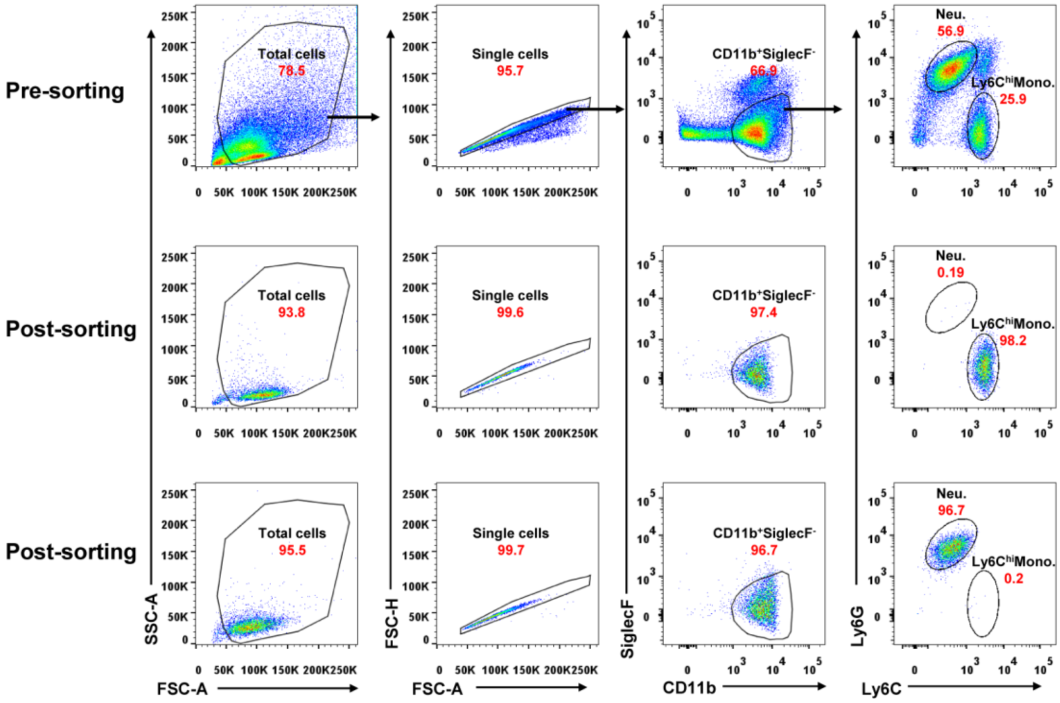

Supplementary Figure 2| Cell sorting strategy and purity of neutrophils and inflammatory monocytes in PLF. WT B6 mice were i.p. injected with OVA/alum, and cells in PLF were collected 4 hours later for sorting. The gating strategy and purity were shown. Inflammatory monocytes in PLF were sorted as CD11b$^+$ SiglecF$^-$Ly6G$^-$Ly6C^{hi} cells, and neutrophils in PLF were sorted as CD11b$^+$ SiglecF$^-$Ly6G^{hi}Ly6C^{low} cells. Neu., neutrophils; Mono., monocytes.

2 Supplementary Table

Supplementary Table 1. Anti-mouse monoclonal antibodies for flow cytometry

Fluorescence	Antigen	Clone	Company	Catalog number	Isotype control	Dilution
APC	CD11c	HL3	BD	550261	ArH IgG1,λ2	1：200
APC-CY7	CD11b	M1/70	BD	557657	Rat IgG2b.κ	1：200
Alexa Fluor® 647	SiglecF	E50-2440	BD	562680	Rat IgG2a, κ	1：200
BV510	MHC-II	M5/114.15.2	BioLegend	107635	Rat IgG2b, κ	1：200
BV605	Ly6C	HK1.4	BioLegend	128035	Rat IgG2c, κ	1：200
BV711	F4/80	BM8	BioLegend	123147	Rat IgG2a, κ	1：200
BV786	CD45	30-F11	BioLegend	103149	Rat IgG2b, κ	1：200
FITC	TLR2	6C2	eB	11-9021	Rat IgG2b,k	1：200
FITC	Ly6C	AL-21	BD	553104	Rat IgM, κ	1：200
FITC	Rat IgG2b,k	A95-1	BD	553988	/	1：200
Pacific Blue	Ly6G	1A8	BioLegend	127611	Rat IgG2a, κ	1：200
PE	SiglecF	E50-2440	BD	552126	Rat IgG2a, κ	1：200
PE	Ly6G	1A8	BD	551461	Rat IgG2a,k	1：200
PE	IL-13	eBio13A	eB	12-7133	Rat IgG1,κ	1：100
PE	CD284(TLR4)	UT41	eB	12-9041-80	Mouse IgG1, κ	1：100
PE	Mouse IgG1, κ	MOPC-31C	BD	550617	/	1：100
PE	Rat IgG1,k	R3-34	BD	554685	/	1：100
PE/Dazzle™ 594	CD3	17A2	BioLegend	100246	Rat IgG2b, κ	1：200
PE-CY7	CD3e	145-2C11	BioLegend	100320	AH IgG	1：200
PE-CY7	CD45	30-F11	BD	552848	Rat IgG2b,k	1：200
PE-CY7	NK1.1	PK136	BD	552878	Ms IgG2a,κ	1：200
Percp-CY5.5	NK1.1	PK136	BioLegend	108728	ms IgG2a,k	1：200
Percp-CY5.5	F4/80	BM8	eB	45-4801	Rat IgG2a,k	1：200
Percp-CY5.5	CD3e	145-2C11	BD	551163	AH IgG1,k	1：200
Percp-CY5.5	CD11b	M1/70	BioLegend	101228	Rat IgG2b,k	1：200
Percp-CY5.5	CD19	6D5	BioLegend	115534	Rat IgG2a,k	1：200
